# Relationship Between Each of the Four Major Motor Symptoms and At-Home Physical Activity in Individuals with Parkinson’s Disease: A Cross-Sectional Study

**DOI:** 10.3390/neurolint17090139

**Published:** 2025-09-03

**Authors:** Yuichi Hirakawa, Hiroaki Sakurai, Kazuya Takeda, Soichiro Koyama, Masanobu Iwai, Ikuo Motoya, Yoshikiyo Kanada, Nobutoshi Kawamura, Mami Kawamura, Shigeo Tanabe

**Affiliations:** 1 Department of Rehabilitation, Kawamura Hospital, 1-84 Daihannya Akutami, Gifu 501-3144, Japan; y.hirakawa2301@gmail.com (Y.H.); masanobuiwai@gmail.com (M.I.); ikuo.m531@gmail.com (I.M.); 2 Graduate School of Health Sciences, Fujita Health University, 1-98 Dengakugakubo, Toyoake 470-1192, Japan; 3 Faculty of Rehabilitation, School of Health Sciences, Fujita Health University, 1-98 Dengakugakubo, Toyoake 470-1192, Japan; hsakurai@fujita-hu.ac.jp (H.S.); kazuya.takeda@fujita-hu.ac.jp (K.T.); koyamas@fujita-hu.ac.jp (S.K.); yokanada@fujita-hu.ac.jp (Y.K.); 4 Department of Neurology, Kawamura Hospital, 1-84 Daihannya Akutami, Gifu 501-3144, Japan; knob0123@gmail.com (N.K.); fmami28@gmail.com (M.K.)

**Keywords:** at-home activity, bradykinesia, Parkinson’s disease, triaxial accelerometer

## Abstract

**Background/Objectives**: Individuals with Parkinson’s disease (PD) often experience four major motor symptoms—tremor, rigidity, bradykinesia, and postural instability/gait disorder. Although these symptoms have been shown to affect activities of daily living, their impact on the level of at-home physical activity (PA) in this population remains unexplored. We aimed to investigate the relationship between the four major motor symptoms of PD and at-home PA in these individuals. **Methods**: This retrospective cross-sectional study included 17 individuals with PD. We examined the relationship between the Movement Disorder Society-sponsored revision of the Unified Parkinson’s Disease Rating Scale Part 3 score and the time spent in three PA intensities (sedentary behavior, light PA [LPA], and moderate-to-vigorous PA) within the home. Pearson’s correlation coefficient was used for statistical analysis. **Results**: In the initial step analysis, a significant negative correlation was observed between the overall motor symptom score and the time spent in LPA inside the home (r_s_ [95% confidence interval]: −0.72 [−0.93 to −0.25]; *p* < 0.01). In the second step analysis, a significant negative correlation was observed between the bradykinesia score and the time spent in LPA inside the home (r_s_: −0.74 [−0.92 to −0.30]; *p* < 0.01). **Conclusions**: Among the four major motor symptoms, only the severity of bradykinesia influenced the time spent in LPA inside the home. Thus, rehabilitation treatment focusing on bradykinesia may be beneficial for increasing the time spent in LPA inside the home for individuals with PD.

## 1. Introduction

Parkinson’s disease (PD) is the second most prevalent progressive neurodegenerative disorder after Alzheimer’s disease [1,2,3]. Between 1990 and 2016, the global prevalence of PD increased markedly from 2.5 million to 6.1 million cases, and projections estimate that this number will exceed 12 million by 2040 [1]. PD develops more frequently in men and in individuals from countries with a medium-to-high sociodemographic index [1].

Although its pathogenesis is not yet fully understood, PD is widely regarded as a multifactorial disease, with approximately 90% of cases considered sporadic and 10% genetically determined [4]. Environmental and lifestyle-related risk factors have also been associated with PD, such as pesticide use, head trauma, tobacco and coffee consumption, and physical inactivity [5].

From a pathological perspective, PD is characterized by the progressive degeneration of dopaminergic neurons in the substantia nigra pars compacta, resulting in dopamine depletion in the basal ganglia [3,6]. A hallmark of the disease is the presence of Lewy bodies, which are intracellular inclusions composed of aggregated α-synuclein filaments [7].

Individuals with PD often experience four major motor symptoms: tremor, rigidity, bradykinesia, and postural instability/gait disorder (PIGD) [8,9,10,11,12,13]. Tremor is classically described as “pill-rolling” (forearm pronation/supination) and is more pronounced in the distal extremities [8,9]. Rigidity is caused by muscular hypertonia, which is independent of velocity and is described as “lead-pipe” resistance, often with the “cogwheel” phenomenon [9,10]. Bradykinesia is characterized by slowing of movement, resulting in reduced speed or amplitude [8,10,11,12]. Additionally, a generalized decrease in spontaneous (e.g., gestures and winking) and deliberate movements (e.g., arm swinging), known as akinesia, has been observed [8,12]. PIGD is an impairment of balance, i.e., impaired ability to maintain or change posture, such as standing and walking [8,13,14,15]. Gait disorders in PD have two typical patterns: festinating gait and freezing of gait.

Previous studies have reported that these four major motor symptoms significantly affect activities of daily living (ADLs) at home [8,16]. For example, resting tremor and rigidity reduce dexterity and affect ADLs such as cutting food, taking medicine, and dressing [9,17]. Bradykinesia slows down movements and reactions and results in a decline in most ADLs, including tasks requiring fine motor control (e.g., buttoning, arranging tableware, and writing) [10,11]. PIGD increases the risk of falls owing to stride length variation and poor balance [9,15].

This decline in ADLs may reduce physical activity (PA) at home. Triaxial accelerometers are widely used as a means to objectively and accurately assess PA in free-living conditions. These devices measure acceleration along three dimensions (*X*, *Y*, and *Z* axes) and are valuable for predicting movement patterns and energy expenditure during various activities [18,19]. They can be used not only to measure PA but also to assist people to increase their PA levels, as they can utilize the behavior change techniques, such as self-monitoring, goal setting, and tailored feedback [20,21]. PA is a multidimensional behavior commonly described using the time spent in three different intensities: sedentary behavior (SB), light PA (LPA), and moderate-to-vigorous PA (MVPA) [22]. Previous studies that evaluated the amount of PA in patients with PD using triaxial accelerometers reported that patients with PD showed a decrease in PA and increased sitting time [23,24,25,26]. Specifically, Pradhan and Kelly [25] investigated PA in individuals with PD and healthy controls over 14 days using a commercially available activity monitor. Their results showed that individuals with PD spent significantly less time in LPA and MVPA and significantly more time in SB compared with age-matched healthy controls [25]. Benka et al. [26] investigated PA in patients with PD over a 7-day period using a triaxial accelerometer. They found that approximately 75% of the awake time was spent in SB, 18% in LPA, and 6% in MVPA in patients with PD [26].

Prior studies have reported a relationship between motor symptoms and PA in individuals with PD [27,28]. Skidmore et al. [27] used the total number of steps as an indicator of PA instead of a triaxial accelerometer and reported a negative correlation between overall motor symptoms and the total number of steps (*p* < 0.05, ρ = −0.48) [27]. However, they assessed overall motor symptoms using the total Movement Disorder Society-sponsored revision of the Unified Parkinson’s Disease Rating Scale (MDS-UPDRS) Part 3 score and did not evaluate each of the four major motor symptoms separately. Additionally, they assessed the total number of steps without differentiating between steps inside and outside the home. To the best of our knowledge, no studies have addressed the relationship between the four major motor symptoms and PA inside the home. Clarifying this relationship would help identify specific impairments that influence PA inside the home and develop effective rehabilitation treatment plans aimed at increasing PA inside the home.

Therefore, this study investigated the relationship between each of the four major motor symptoms and at-home PA in individuals with PD.

## 2. Materials and Methods

### 2.1. Study Design and Participants

This study was conducted following the guidelines of the International Committee of Medical Journal Editors and the Declaration of Helsinki. The study was approved by the Human Ethics Committee of Fujita Health University (approval number: HM22-198). The requirement for written informed consent was waived owing to the retrospective observational design. However, to provide participants with the opportunity to decline the use of their health records for the study, we posted study information with an opt-out option on our hospital’s bulletin board. The study is reported following the Strengthening the Reporting of Observational Studies in Epidemiology statement [29].

This retrospective, cross-sectional, single-center study included outpatients evaluated at Kawamura Hospital between October 2022 and December 2024 selected through convenience sampling. The inclusion criteria were as follows: (1) PD diagnosed by a neurologist, (2) PD classified as Hoehn and Yahr (HY) stages 1 to 4, and (3) ability to walk independently with or without assistive devices. The following were the exclusion criteria: (1) engagement in work outside the home at the time of study participation, (2) uncontrolled diabetes or hypertension, (3) chronic obstructive pulmonary disease, (4) history of coronary artery disease or congestive heart failure, (5) history of neuromuscular disease other than PD, and (6) missing data.

### 2.2. Demographic and Clinical Characteristics

We collected the following demographic and clinical data: sex, age, time since PD diagnosis, levodopa equivalent daily dose (LEDD), and HY stage. Levodopa is an antiparkinsonian drug that remains the basis of pharmacological treatment of PD. However, higher doses of levodopa, and possibly other drugs, produce better symptom control but more delayed complications. To address this problem, conversion factors have been calculated for antiparkinsonian drugs that yield a total daily LEDD [30]. The HY stage was evaluated to determine the disease severity [31]. The HY stage was determined based on the five-item HY scale, as follows: 1 = Unilateral involvement only, usually with minimal or no functional disability; 2 = Bilateral or midline involvement without impairment of balance; 3 = Bilateral disease: mild to moderate disability with impaired postural reflexes; physically independent; 4 = Severely disabling disease; still able to walk or stand unassisted; and 5 = Confinement to bed or wheelchair unless aided [31].

### 2.3. At-Home PA Measurement

Based on the method used in a previous study [32], we assessed at-home PA using a combination of triaxial accelerometer measurements and a daily activity diary. Specifically, the participants wore a triaxial accelerometer on their waist during waking hours for seven consecutive days and simultaneously recorded their at-home daily activities in a diary, which enabled the assessment of time spent at different intensities of PA inside the home [32].

PA was measured using the Active-style Pro HJA-750C triaxial accelerometer (Omron Healthcare, Kyoto, Japan [width 40 mm  ×  height 52 mm  ×  depth 12  mm, weight 23.0  g]). The anteroposterior (*x*-axis), mediolateral (*y*-axis), and vertical (*z*-axis) acceleration signals were collected at 32 Hz. In addition, the triaxial accelerometer recorded activity levels, which were expressed as metabolic equivalents (METs) per minute [33]. MET data were estimated in 10 s epochs and output every minute [33]. Regarding the estimated accuracy of METs, a previous study reported a significant positive correlation between energy expenditure during physical activity measured by Active Style Pro and doubly labeled water (r = −0.46, *p* = 0.04) [34]. The participants were asked to wear the accelerometer on their waist for at least 7 days from the time they woke up until they went to bed, except during water-based activities (e.g., bathing, showering, and swimming) [34,35]. This study set the monitoring period to seven consecutive days because this duration has been widely adopted to account for day-to-day variability, including both weekdays and weekends [36,37,38]. The triaxial accelerometer recorded activity levels, which were expressed as metabolic equivalents (METs) per minute [33]. Based on METs, the obtained data were divided into three PA intensities: SB was defined as 1.0 < METs ≤ 1.5, LPA as 1.5 < METs < 3.0, and MVPA as ≥3.0 METs [27]. The time spent in each PA intensity per day and the mean values over 7 days were calculated.

Daily activity diaries were used to extract at-home PA information [32]. The daily activity diary was recorded at 10 min intervals as time steps, starting from waking up and ending with going to bed. The participants completed the daily activity diary in their own words across three domains: time of day (e.g., 7:00–8:00 a.m./p.m.), activity (e.g., having breakfast, dressing, brushing teeth, washing one’s face, cleaning, doing laundry, and shopping), and location (outdoor or indoor). They could also enter a free description (forgetting to wear a PA meter, poor health, taking medicine, occurrence of dyskinesia, and on-off time). When going out, the participants were asked to specify the mode of transportation used (e.g., walking, private car, or bus). To reduce the burden incurred by completing the diary at once, the participants completed the daily activity diary for 1 day in two batches: after lunch and before going to bed [32].

### 2.4. Motor Symptom Measurement

The MDS-UPDRS Part 3 was used to quantify the overall and individual motor symptoms: tremor, rigidity, bradykinesia, and PIGD [39]. The MDS-UPDRS has high internal consistency (Cronbach’s alpha [α] = 0.93) and strong concurrent validity in individuals with PD [39]. This tool comprises 33 items based on 18 questions, where the evaluator explains the tasks to the participants and instructs their performance. The questions in MDS-UPDRS Part 3 were rated on a five-point scale, as follows: 0 points (normal performance), 1 point (slight impairment), 2 points (mild impairment), 3 points (moderate impairment), and 4 points (severe impairment) [39]. The maximum score for the MDS-UPDRS Part 3 is 132 points, with higher total scores indicating greater severity of overall motor symptoms. Additionally, we used definitions reported in a previous study to calculate the scores for each of the four major motor symptoms: tremor (3.15–3.18), rigidity (3.3a–3.3e), bradykinesia (3.4–3.8 and 3.14), and PIGD (3.9–3.13) [40]. Specifically, tremor was assessed using the following items (3.15–3.18): Postural tremor of the hands, Kinetic tremor of the hands, Rest tremor amplitude, and Constancy of rest tremor. Rigidity was assessed using the following items (3.3a–3.3e): Rigidity–neck, Rigidity–right upper extremity, Rigidity–left upper extremity, Rigidity–right lower extremity, and Rigidity–left lower extremity. Bradykinesia was assessed using the following items (3.4–3.8 and 3.14): Finger tapping–right hand, Finger tapping–left hand, Hand movements–right hand, Hand movements–left hand, Pronation–supination movements–right hand, Pronation–supination movements–left hand, Toe tapping–right foot, Toe tapping–left foot, Leg agility–right leg, Leg agility–left leg, and Global spontaneity of movement. PIGD was assessed using the following items (3.9–3.13): Arising from chair, Gait, Freezing of gait, Postural stability, and Posture [39,40].

### 2.5. Statistical Analysis

The overall motor symptom score (i.e., total MDS-UPDRS Part 3 score), individual motor symptom scores, and the time spent in three different PA intensities (i.e., SB, LPA, and MVPA) inside the home were calculated for each participant. As a first step in the analysis, we investigated the relationship between the overall motor symptom score and time spent in SB, LPA, and MVPA at home. In the second step, we investigated the relationship between each of the four major motor symptom scores and the PA intensity item that correlated with the overall motor symptom score in the first step.

The Shapiro–Wilk normality test was used to test the data distribution. Normally distributed data were analyzed using Pearson’s correlation coefficient, whereas non-normally distributed data were analyzed using Spearman’s rank correlation. The correlations were interpreted as low (<0.25), fair (from 0.25 to <0.5), moderate to good (from 0.5 to <0.75), and good to excellent (greater than or equal to 0.75) [41]. Outliers were analyzed using both the Z-score and the interquartile range (IQR) methods. A Z-score greater than 3.0 or a value outside the range of Q1 − 1.5 × IQR to Q3 + 1.5 × IQR indicated an outlier [42,43]. As a result, one outlier was identified in a single variable (tremor). Because the results of the analyses with and without the outlier were consistent, we included the outlier in the final analysis. Statistical significance was set at *p* < 0.01. All statistical analyses were performed using EZR version 1.68 (EZR Saitama Medical Center, Jichi Medical University, Saitama, Japan).

## 3. Results

**Figure 1:**
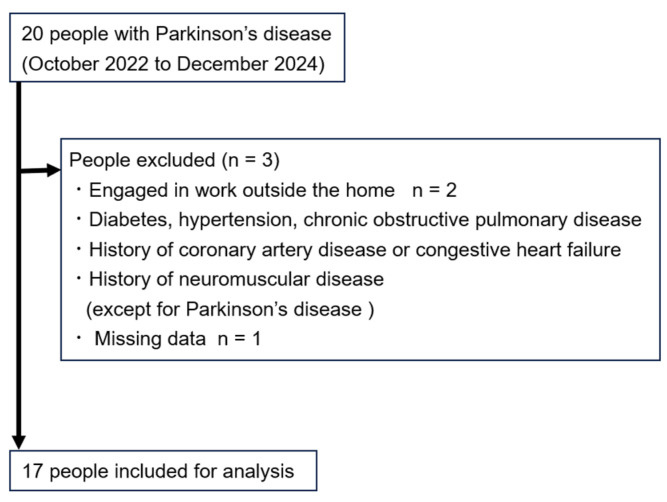
Flowchart of participant enrolment.

We recruited 17 eligible individuals with PD from among 20 patients (Figure 1). Their clinical characteristics are presented in Table 1. Participants spent 81.2 ± 15.1% of the time wearing the triaxial accelerometer inside the home and 18.8 ± 16.8% outside the home. Participants spent 77.8 ± 6.1% of their time at home in SB, 21.1 ± 6.1% in LPA, and 1.1 ± 0.9% in MVPA.

**Figure 2:**
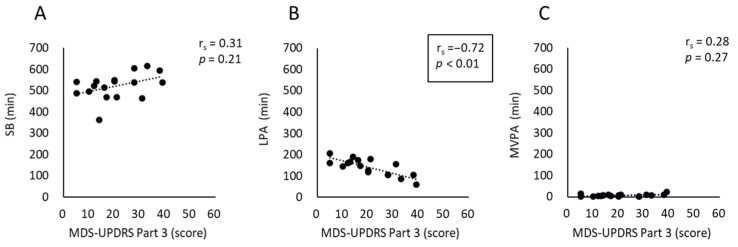
Scatterplot and linear trend line showing Spearman’s rank correlation between total MDS-UPDRS Part 3 score and time spent in three PA intensities inside the home: (**A**) SB, (**B**) LPA, and (**C**) MVPA. The box indicates statistically significant correlations (*p* < 0.01). MDS-UPDRS, Movement Disorder Society-sponsored revision of the Unified Parkinson’s Disease Rating Scale; SB, sedentary behavior; LPA, light physical activity; MVPA, moderate-to-vigorous-intensity physical activity.

Figure 2 shows the results of Spearman’s rank correlation analyses examining the relationship between the overall motor symptom score and the time spent in the three PA intensities inside the home. The total MDS-UPDRS Part 3 score was significantly correlated with the time spent in LPA inside the home (r_s_ [95% confidence interval {95% CI}] = −0.72 [−0.93 to −0.25], *p* < 0.01), with no significant correlations with the time spent in SB or MVPA inside the home (r_s_ [95% CI] = 0.31 [−0.22 to 0.66], *p* = 0.21 and r_s_ [95% CI] = 0.28 [−0.30 to 0.74], *p* = 0.27, respectively).

**Figure 3:**
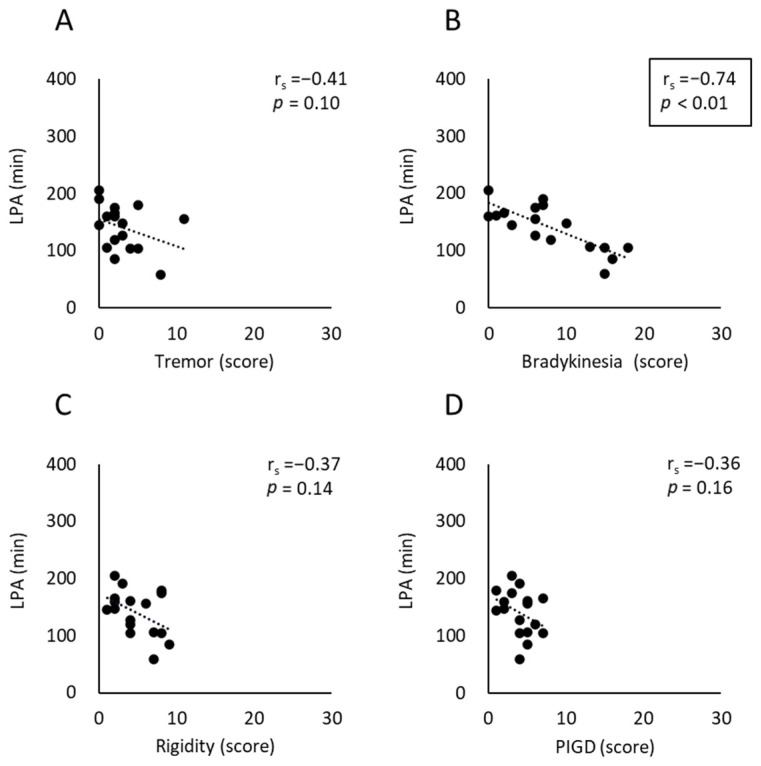
Scatterplot and linear trend line showing Spearman’s rank correlation between each of the four major motor symptom scores of PD—(**A**) tremor, (**B**) rigidity, (**C**) bradykinesia, and (**D**) PIGD, calculated from the MDS-UPDRS Part 3—and the time spent in LPA inside the home. The box indicates statistically significant correlations (*p* < 0.01). In (**A**) tremor, one outlier was identified. Because the results of the analyses with and without the outlier were consistent, the outlier was included in the final analysis. LPA, light physical activity; PIGD, postural instability/gait disorder.

The results of Spearman’s rank correlation analyses between each of the four major motor symptoms and the time spent in LPA inside the home are shown in Figure 3. The bradykinesia score was significantly correlated with the time spent in LPA inside the home (r_s_ [95% CI] = −0.74 [−0.92 to −0.30], *p* < 0.01), whereas the tremor, rigidity, and PIGD scores showed no significant correlation with the time spent in LPA inside the home (r_s_ [95% CI] = −0.41 [−0.78 to 0.12], *p* = 0.10; r_s_ [95% CI] = −0.37 [−0.79 to 0.17], *p* = 0.14; and r_s_ [95% CI] = −0.36 [−0.67 to 0.06], *p* = 0.16, respectively).

## 4. Discussion

This study investigated the relationship of overall motor symptoms and each of the four major motor symptoms with at-home PA in individuals with PD. We observed a significant negative correlation between the overall motor symptom score and the time spent in LPA inside the home. Additionally, a significant negative correlation was observed between the bradykinesia score and the time spent in LPA inside the home.

The severity of overall motor symptoms did not influence SB or MVPA. This finding is consistent with the findings of previous studies not limited to at-home PA [24,25,26], which reported that individuals with PD spend most of their day inactively regardless of the severity of their motor symptoms. Even individuals with mild to moderate motor symptoms were classified under SB [24,25,26], and most individuals performed little MVPA [24,25]. For example, Pradhan and Kelly [25] reported that individuals with mild PD spend only 2.7% of their awake time on MVPA.

The severity of overall motor symptoms influenced the time spent in LPA inside the home. Furthermore, among the four major motor symptoms, only the severity of bradykinesia influenced the time spent in LPA. A previous study reported that LPA includes standing and walking activities with slow upper-body movements [44]. Lana et al. [45] investigated the main factors influencing PA levels in individuals with PD and reported that lower-limb bradykinesia was a predictor of PA levels in this population. In terms of ADLs, activities classified as LPA include housework, grooming, and indoor gardening [44]. Previous studies reported that bradykinesia influences tasks such as arranging tableware during housework, buttoning during dressing, and walking by reducing the speed or amplitude of movements in the upper and lower limbs [8,10,11]. In contrast, our findings suggest that the severity of tremor, rigidity, and PIGD may not influence time spent in various PAs inside the home classified as LPA. Tremor and rigidity can reduce dexterity in limb movements. For example, when tremor and rigidity in the upper limbs are worsened, they affect the performance of fine motor tasks such as cutting food performed while seated and opening medication packages [2,10]. PIGD is strongly associated with balance impairments that can cause difficulty in maintaining or changing posture [2,8].

The findings from this study suggest that rehabilitation interventions targeting bradykinesia may be important for increasing the time spent in LPA inside the home by individuals with PD. Bouça-Machado et al. [45] reported significant health benefits in individuals with PD by replacing the time spent in SB with LPA. Numerous studies have evaluated rehabilitation interventions and their effectiveness in bradykinesia treatment [46,47,48,49,50]. Iwai et al. [47] investigated the effectiveness of Lee Silverman Voice Treatment BIG (LSVT^®^ BIG) therapy in individuals with mild-to-moderate PD. LSVT BIG incorporates external cueing and task-based interventions and consists of four parts: (1) standardized whole-body exercise, (2) functional tasks, (3) hierarchical tasks, and (4) BIG walking. Each session lasted 1 h, four times a week, for 4 weeks. After 4 weeks of LSVT^®^ BIG therapy intervention, the authors observed a significant improvement in bradykinesia scores extracted from the MDS-UPDRS Part 3 [46]. Dibble et al. [49] investigated the effects of high-force eccentric resistance exercises on muscle weakness, which contributed to bradykinesia. The exercise program included high-intensity quadriceps contractions on an eccentric ergometer, performed thrice a week for 12 weeks. The results showed improvements in clinical bradykinesia measures (gait speed and timed up-and-go test) [49].

This study has some limitations. First, owing to the single-center, retrospective, cross-sectional design, the generalizability of the findings to a broader population of individuals with PD may be limited. In addition, we did not perform analyses using the data of only male and only female participants. Given the limited number of participants, we did not aim to examine the influence of sex on the relationship between motor symptoms and PA inside the home. The supplementary analysis stratifying the participants based on sex yielded results that differed from those of our main findings (Tables S1 and S2); however, as the number of participants in each subgroup was small, these results should be interpreted with caution. Furthermore, we did not perform a stratified analysis based on disease progression (i.e., HY stage). Therefore, to verify our results, multicenter studies involving a larger number of participants and stratified analyses (i.e., sex and HY stage) are needed. Second, given the study design, we were unable to establish a causal relationship between motor symptoms and at-home PA in individuals with PD. To determine whether improvements in bradykinesia lead to increased time spent in LPA inside the home, longitudinal or interventional studies are required. Third, this study assessed motor symptoms using the MDS-UPDRS Part 3, which is the gold standard for clinical evaluation. Using this scale, we identified an association between the severity of bradykinesia and LPA inside the home. Nevertheless, additional quantitative metrics may provide a more detailed assessment. For example, Memar et al. reported that whole-body movement could be quantified using wearable inertial measurement units and proposed a bradykinesia index based on this approach [50]. Despite these limitations, our findings provide important preliminary evidence that bradykinesia influences the time spent in LPA inside the home in individuals with PD.

## 5. Conclusions

We observed a significant negative correlation between the overall motor symptom score and the time spent in LPA inside the home, whereas SB and MVPA were not significantly influenced by motor symptom severity. Additionally, among the four major motor symptoms, only the severity of bradykinesia influenced the time spent in LPA inside the home. Our findings may help select treatment modalities for individuals with PD; specifically, rehabilitation treatment focusing on bradykinesia may be beneficial for increasing the time spent in LPA inside the home.

## Figures and Tables

**Table 1:** Demographic and clinical characteristics of the participants.

Sex [M/F]	No.	Age [Years]	MMSE [Points]	Time Since Diagnosis [Years]	LEDD [mg/Day]	HY Stage	SB [min]	LPA [min]	MVPA [min]	MDS-UPDRS Part 3 [Scores]	Tremor	Rigidity	Bradykinesia	PIGD
F	1	78	30	11	650	2	470.1	147.8	5.1	17	3	2	10	2
	2	63	30	5	150	2	515.7	175.0	10.6	16	2	8	6	3
	3	86	29	7	900	4	542.6	119.3	6.4	20	2	4	8	6
	4	64	30	8	100	2	488.6	160.0	2.1	5	1	2	0	2
	5	53	30	2	600	2	468.6	180.0	10.6	21	5	8	7	1
	6	57	29	4	350	2	522.9	160.6	4.0	12	2	4	1	5
	7	71	25	8	400	3	544.6	165.6	3.6	13	2	2	2	7
	8	63	30	10	500	4	538.7	58.7	24.3	39	8	7	15	4
	9	70	27	0	0	2	463.6	155.7	10.7	31	11	6	6	5
	10	66	29	0	0	2	363.6	190.9	8.4	14	0	3	7	4
	Subtotal	67.1 (9.2)	29.5 [28.5, 30.0]	5.5 (3.7)	365.0 (287.3)	2.0 [2.0, 4.0]	491.9 (52.3)	151.3 (36.0)	8.6 (6.0)	16.5 [12.8, 23.5]	2.0 [1.8, 5.8]	4.0 [2.0, 7.3]	6.5 [1.8, 8.5]	4.0 [2.0, 5.3]
M	11	75	25	2	350	2	604.3	104.3	3.0	28	4	4	15	4
	12	80	27	3	600	3	594.3	104.3	10.0	38	5	8	18	7
	13	76	20	5	400	4	615.7	85.0	7.6	33	2	9	16	5
	14	47	30	17	450	1	541.0	205.7	16.0	5	0	2	0	3
	15	74	23	3	300	3	538.8	105.8	1.4	28	1	7	13	5
	16	72	30	3	400	4	550.0	126.8	1.8	20	3	4	6	4
	17	73	25	3	380	4	497.0	145.0	2.2	10	0	1	3	1
	Subtotal	71.0 (10.1)	25.0 [23.0, 30.0]	5.1 (4.9)	411.4 (88.2)	3.0 [2.0, 4.0]	563 (39.7)	125 (37.3)	6.0 (5.1)	28.0 [10.0, 33.0]	2.0 [0, 4.0]	4.0 [2.0, 8.0]	13.0 [3.0, 16.0]	4.0 [3.0, 5.0]
	Grand total	68.7 (9.8)	29.0 [25.0, 30.0]	5.4 (4.3)	384.1 (228.6)	2.0 [2.0, 4.0]	521.2 (59.0)	140.6 (38.7)	7.5 (5.8)	20.0 [12.5, 29.5]	2.0 [1.0, 4.5]	4.0 [2.0, 7.5]	7.0 [2.5, 14.0]	4.0 [2.5, 5.0]

The data are median [interquartile range] or mean (standard deviation). LEDD, levodopa equivalent daily dose; HY, Hoehn and Yahr; SB, sedentary behavior; LPA, light physical activity; MVPA, moderate-to-vigorous physical activity; MDS-UPDRS, Movement Disorder Society-sponsored revision of the Unified Parkinson’s Disease Rating Scale; PIGD, postural instability/gait disorder.

## Data Availability

Part of the data presented in this study is available in Supplementary Materials. Other data supporting the findings of this study are available from the corresponding author upon reasonable request.

## Supplementary Materials

The following supporting information can be downloaded at: https://www.mdpi.com/article/10.3390/neurolint17090139/s1, Table S1: Relationship between the total MDS-UPDRS Part 3 score and the time spent in three PA intensities inside the home in males (*n* = 7); Table S2: Relationship between the total MDS-UPDRS Part 3 score and the time spent in three PA intensities inside the home in females (*n* = 10).

## Abbreviations

The following abbreviations are used in this manuscript:

ADLs	Activities of daily living
CI	Confidence interval
HY	Hoehn and Yahr
LPA	Light physical activity
LEDD	Levodopa equivalent daily dose
LSVT BIG	Lee Silverman Voice Treatment BIG
MDS-UPDRS	Movement Disorder Society-sponsored revision of the Unified Parkinson’s Disease Rating Scale
MET	Metabolic equivalent
MVPA	Moderate-to-vigorous physical activity
PA	Physical activity
PD	Parkinson’s disease
PIGD	Postural instability/gait disorder
SB	sedentary behavior
