# Supplementary material for: Relationship Between Each of the Four Major Motor Symptoms and At-Home Physical Activity in Individuals with Parkinson’s Disease: A Cross-Sectional Study
Source: Neurol Int. 2025 Sep 3;17(9):139. doi: 10.3390/neurolint17090139 (PMC12472460; doi:10.3390/neurolint17090139)
Supplement: Supplementary file 1 [file neurolint-17-00139-s001.zip › neurolint-3784774_Supplementary_Files_S2.pdf]

Table S2. Relationship between the total MDS-UPDRS Part 3 score and the time spent in three PA intensities inside the home in females (n=10).

	Spearman's rank correlation coefficient	p-value
SB	-0.127	0.733
LPA	-0.418	0.232
MVPA	0.875	<0.001
